# Isolated femoral nerve neurofibroma with vastus medialis muscle atrophy as the initial symptom: a case report and literature review

**DOI:** 10.3389/fonc.2026.1814048

**Published:** 2026-06-03

**Authors:** Xinxin Liu, Ying Lu, Yiqi Huang

**Affiliations:** 1 Electromyography room of Department of Functional Examination, Tongde Hospital of Zhejiang Province, Hangzhou, Zhejiang, China; 2 Department of Nephrology, Shaoxing Second Hospital, Shaoxing, Zhejiang, China

**Keywords:** case report, isolated femoral nerve neurofibroma, literature review, the initial symptom, vastus medialis muscle atrophy

## Abstract

**Background:**

Neurofibromas are benign tumors that originate in the peripheral nerve sheath. Most patients initially present with a palpable mass on the body. In this patient, no body surface mass was detected, and the initial symptoms were muscle pain and atrophy. Isolated femoral neurofibromas have been reported less frequently.

**Case presentation:**

In June 2024, a 38-year-old man was admitted to the hospital due to pain in his left knee and the inner side of his thigh for 2–3 months, accompanied by muscle atrophy for half a month. After electromyography, ultrasound examination of the thigh muscles, and enhanced MR scan of the femur, and through a multidisciplinary case discussion, a high suspicion of femoral nerve fibroma was formed. At another hospital, the “left femoral nerve tumor resection + nerve transplantation + pedicled composite tissue flap formation + gastrocnemius nerve resection” surgery was performed, and the postoperative pathology confirmed it to be a neurofibroma.

**Conclusion:**

Most neurofibromas initially present as visible masses on the body. However, there are also cases in which no body surface mass is detectable, and the initial symptoms may be muscle pain and muscle atrophy. Because these tumors are prone to recurrence, thorough clinical and imaging examinations, as well as long-term follow-up, are necessary.

## 1 Introduction

Neurofibromas are benign tumors that originate from the peripheral nerve sheath and can occur in any tissue or organ containing nerve fibers (1–3). It is most commonly found in areas such as the neck and limbs (4). According to the World Health Organization, neurofibromas are benign tumors characterized by a peripheral nerve sheath phenotype with mixed cellular components, including Schwann cells, perineurial hybrid cells, and perineurial fibroblasts (5). It constitutes approximately 5% of all benign soft tissue tumors and typically manifests as a painless, slow-growing mass (6).

Most neurofibromas present as palpable skin masses, which are typically the initial symptom. However, in this patient, the femoral neurofibroma did not present with a palpable skin mass; instead, the initial symptoms were muscle pain and atrophy. This case serves as a reminder to clinicians that patients presenting with lower limb muscle atrophy and pain may have neurogenic tumors. Early diagnosis can help prevent misdiagnosis, avoid worsening of the condition, and provide timely treatment.

## 2 Case presentation

On June 18, 2024, a 38-year-old male was admitted to the neurology department of our hospital with pain in the left knee and medial thigh for 2–3 months, accompanied by muscle atrophy that had developed over the preceding two weeks.

Approximately three months before admission, the patient experienced sudden onset of left knee pain without an obvious trigger, which gradually radiated to the medial thigh and was mainly described as soreness. Approximately two weeks before admission, muscle atrophy of the medial thigh became apparent.

On June 11, 2024, prior to admission, lumbar computed tomography (CT) revealed degeneration, disc protrusion at the L4/L5 level, and mild osteophytic changes. On June 13, 2024, MRI of the left femur revealed a small amount of fluid between the rectus femoris and vastus medialis muscles. The patient received topical analgesics and blood-activating therapy; however, symptoms did not improve significantly, and he continued to experience medial thigh and mild lower back pain, prompting hospitalization for further evaluation.

On admission, physical examination revealed a body temperature of 37.1 °C and blood pressure of 125/82 mmHg. The muscle strength was normal (grade 5) in all limbs. However, atrophy of the left medial thigh muscle group was observed, with a reduced circumference of the left lower leg compared to that of the right leg. Tendon reflexes were (+++) in the left upper limb and both lower limbs and (++) in the right upper limb.

**Figure 1:**
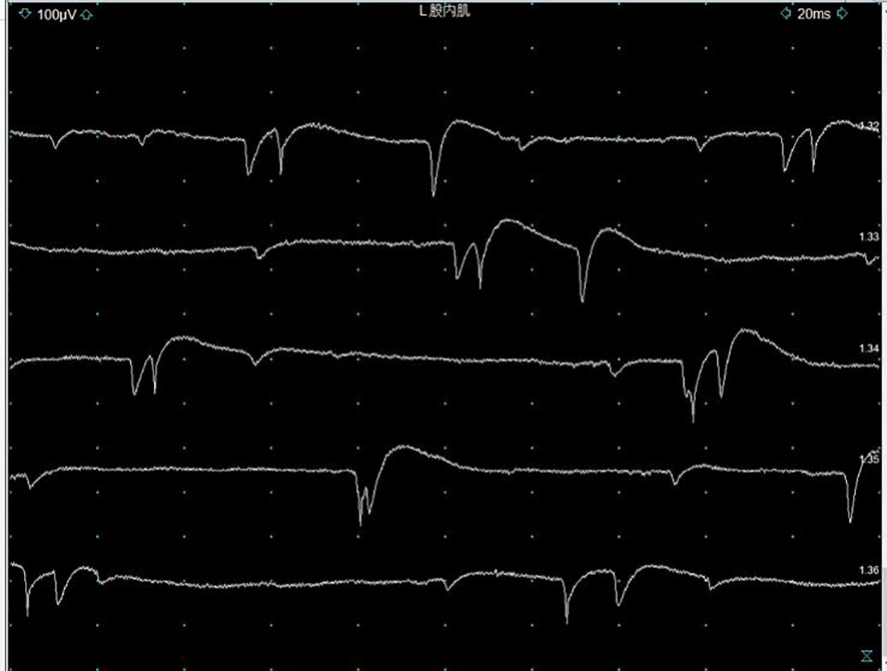
Needle electromyography of the left vastus medialis muscle showing spontaneous activity (+) at rest, indicating abnormal spontaneous muscle activity.

On June 18, 2024, electromyography (EMG) demonstrated neurogenic damage in the left vastus medialis muscle with no obvious abnormalities in the vastus lateralis muscle. Spontaneous activity (+) was observed in the left vastus medialis muscle at rest (**Figure 1**).

**Figure 2:**
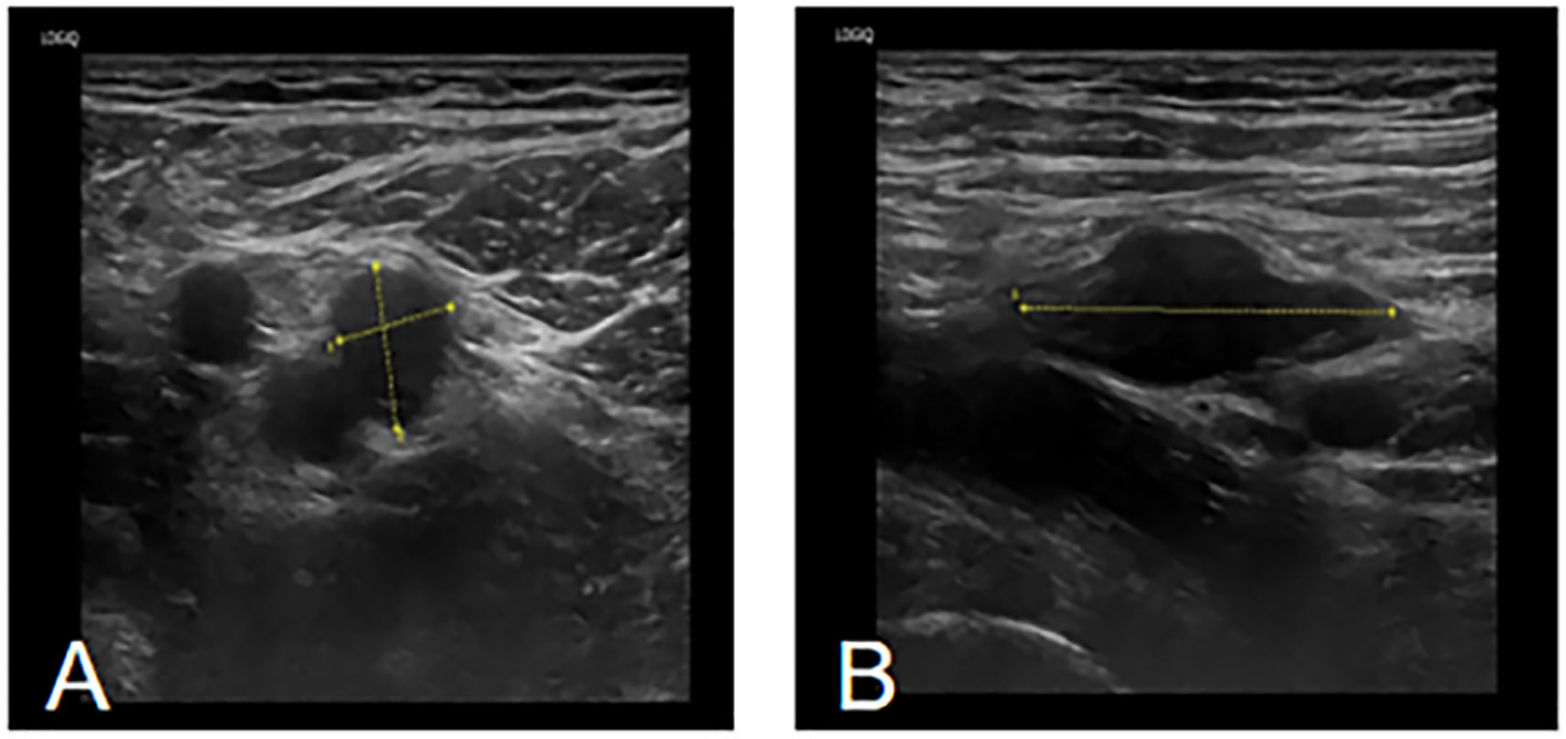
Ultrasound findings of the left thigh muscles and inguinal region. **(A)** Transverse grayscale ultrasound image showing a well-defined, homogeneously hypoechoic lesion adjacent to the left femoral artery. **(B)** Longitudinal grayscale ultrasound image of the same lesion demonstrating its configuration along the femoral nerve. No significant internal blood flow was detected on CDFI. Mild atrophy of the left thigh muscles was observed compared with the contralateral side. These findings are consistent with a probable lesion of femoral nerve origin, most likely a neurofibroma.

On June 19, ultrasound examination of the left thigh revealed mild atrophy of the left thigh muscles compared to the contralateral side. A well-defined hypoechoic mass measuring approximately 3.0 × 1.0 × 1.0 cm was identified in the left inguinal region, closely associated with the femoral nerve. These findings suggested a hypoechoic mass in the left inguinal region, likely arising from the femoral nerve. Neurofibroma was considered the most probable diagnosis (**Figure 2**).

**Figure 3:**
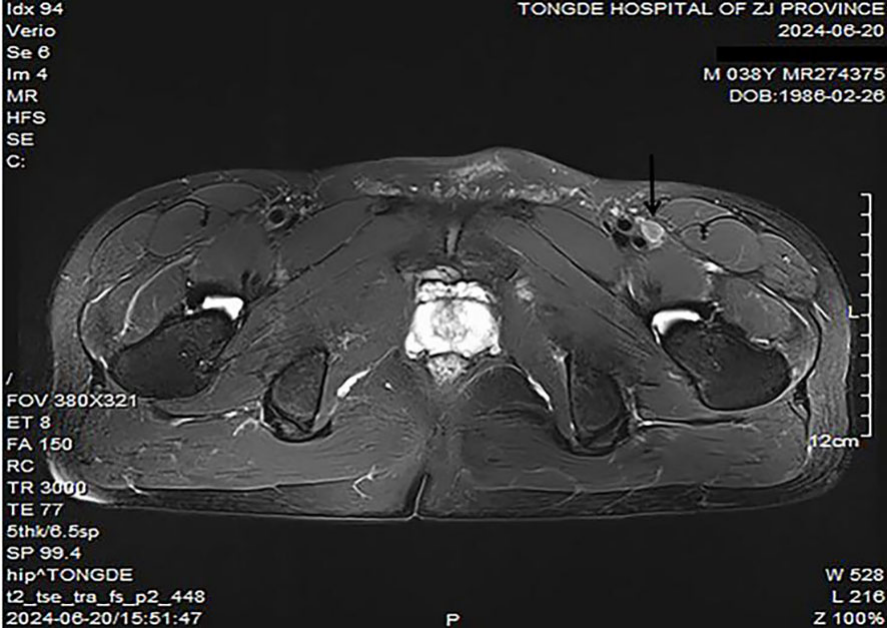
Contrast-enhanced MRI of the left femoral region. A fusiform lesion measuring approximately 3.0 × 2.0 × 1.0 cm is observed adjacent to the artery in the left inguinal region. The lesion shows an isointense signal on T1-weighted imaging and a slightly hyperintense signal on T2-weighted imaging, with well-defined margins. Following contrast administration, it demonstrates moderate enhancement with relatively weaker internal enhancement. The lesion is suggestive of a tumor of probable neural origin. The arrow indicates the lesion.

On June 20, contrast-enhanced MRI of the left femoral region demonstrated a fusiform lesion measuring approximately 3.0 × 2.0 × 1.0 cm in the left inguinal region. The lesion showed an isointense signal on T1-weighted images and a slightly hyperintense signal on T2-weighted images with well-defined margins. Moderate enhancement was observed after contrast administration, with relatively weak internal enhancement, suggesting a neurogenic tumor as the most likely diagnosis (**Figure 3**).

On June 25, 2024, following a multidisciplinary discussion, surgical treatment involving nerve tumor resection and possible nerve transplantation was recommended. The risks and costs were explained; however, the patient declined treatment at our hospital and requested discharge to seek care elsewhere.

**Figure 4:**
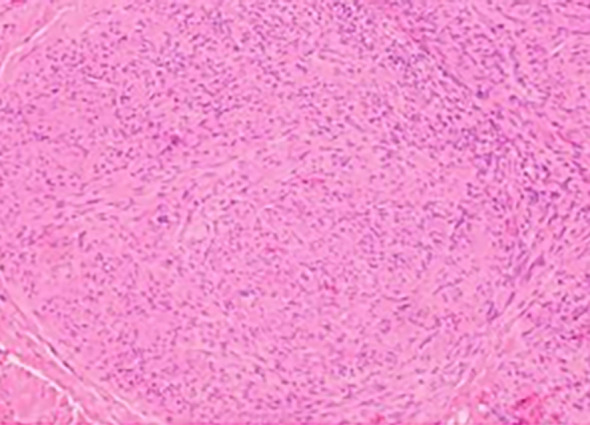
Microscopic findings of postoperative pathology.

After excluding surgical contraindications, the patient underwent surgery at another institution on September 28, 2024, including resection of the left femoral nerve tumor, nerve transplantation, pedicle composite tissue flap reconstruction, and sural nerve neurotomy. Postoperative pathological examination confirmed the diagnosis of neurofibroma (**Figure 4**).

On November 11, 2024, during follow-up, the patient reported improvement in muscle atrophy compared to the preoperative status; however, complete sensory loss persisted in the saphenous nerve distribution on the medial aspect of the lower leg.

On January 22, 2025, follow-up electromyography (EMG) demonstrated incomplete injury to the left femoral and saphenous nerves, with an absent sensory nerve action potential in the left sural nerve despite repeated stimulation.

By August 14, 2025, the patient showed marked recovery, with significant improvement in muscle atrophy and substantial relief of numbness on the medial side of the calf.

## 3 Discussion and conclusion

Neurofibromas are typically multifocal lesions associated with neurofibromatosis resulting from inactivation or mutation of the NF-1 gene located on the long arm of chromosome 17 (5, 7, 8). This condition is commonly referred to as type 1 neurofibromatosis (NF-1) or von Recklinghausen’s disease (VRD). Worldwide, the prevalence of NF-1 is approximately 1 in every 3,000 people (9), which can cause skin changes and bone deformities and can present as isolated lesions or as part of the more extensive disease of neurofibromatosis (8). In this case, vastus medialis muscle atrophy was attributed to solitary femoral nerve neurofibroma (10). Although neurofibromas can be solitary or multifocal, schwannomas are the most common type of solitary tumor, and multiple tumors are characteristic of neurofibromatosis (11). Isolated neurofibromas are more commonly found in the head and neck region, as well as the flexural sides of the limbs, with a typical distribution in the cervical,> vagus,> peroneal> ulnar nerves. A literature review revealed that isolated femoral nerve neurofibromas have rarely been reported.

Neurofibroma is a histological lesion originating from the connective tissue of the sheath within the peripheral nerves that typically affects ectodermal organs. They comprise a mixture of Schwann cells, fibroblasts, and mast cells (12). Surgical treatment is the most effective method for controlling or curing this condition (13, 14). The distinction between neurofibromas and schwannomas lies in two key differences.: ① Neurofibromas are composed of various cell types, whereas schwannomas are predominantly composed of well-differentiated Schwann cells (15). ② Neurofibromas typically involve the nerve endoneurium and are closely integrated with the parent nerve, making complete excision challenging. In the present case, intraoperative findings confirmed that the tumor involved the endoneurium and was tightly associated with the affected nerve. In contrast, schwannomas are usually encapsulated and eccentrically located relative to the parent nerve, allowing easier surgical separation with less risk of nerve injury (16). Total local resection is the preferred treatment for benign peripheral nerve tumors. Distinguishing between schwannomas and neurofibromas is crucial for determining the most appropriate surgical approach. Because schwannomas have well-defined capsules, they can be surgically removed while preserving nerve continuity. However, the removal of neurofibromas is more challenging because they lack a capsule and are diffusely spread, often resulting in nerve damage. Additionally, neurofibromas tend to spread more extensively than schwannomas, requiring more extensive surgical removal (17) and, in some cases, nerve grafts, such as sural nerve transplants. The recurrence rate after surgical removal of neurofibromas is low, and the risk of malignant transformation ranges from 5% to 10%, particularly in patients with NF-1. The patient’s postoperative pathological diagnosis confirmed a neurofibroma, and the prognosis was favorable with follow-up care.

As a neurofibroma enlarges or undergoes cystic transformation, it begins to compress and irritate the surrounding nerves. Clinical manifestations include aching sensations along the nerve distribution area, abnormal sensations, loss of sensation in the affected area, and muscle atrophy (1). In this patient with femoral nerve neurofibroma, the initial symptoms were pain and muscle atrophy of the vastus medialis innervation. MRI is the preferred imaging modality for evaluating peripheral nerve lesions, as it helps differentiate between schwannomas, neurofibromas, and other soft tissue tumors (18).

In addition to schwannomas and other soft tissue tumors, post-traumatic neuroma should be considered in the differential diagnosis of focal peripheral nerve lesions. Posttraumatic neuromas are non-neoplastic proliferations that arise from disorganized nerve regeneration following injury, and are often associated with a history of trauma or surgery (19).

Imaging plays a crucial role in distinguishing these entities. On ultrasonography, traumatic neuromas typically appear as hypoechoic and irregular enlargements of the affected nerve, sometimes with disruption of normal nerve continuity (20). On MRI, they usually present as focal nerve thickening with increased T2-weighted signal intensity and may be accompanied by surrounding soft tissue edema or denervation changes (21). However, considerable overlap exists in the imaging characteristics of traumatic neuromas and peripheral nerve sheath tumors, which limits the diagnostic specificity of conventional imaging modalities, such as ultrasound and MRI (22–24).

For example, Atik et al. (19). reported sonographic and MRI findings of a post-traumatic median nerve neuroma, emphasizing the importance of identifying nerve continuity changes and correlating imaging findings with clinical history to achieve an accurate diagnosis. Compared to traumatic neuromas, neurofibromas are true neoplastic lesions that are not necessarily associated with prior injury and tend to present as more homogeneous fusiform masses along the course of the nerve. Traumatic neuromas are typically associated with a history of nerve injury and often demonstrate irregular morphology and disrupted nerve continuity, whereas neurofibromas usually present as homogeneous fusiform lesions without a clear history of trauma. In the present case, the absence of a history of trauma, together with the pathological findings, supported the diagnosis of a neurofibroma rather than a post-traumatic neuroma.

Electromyography (EMG) is a diagnostic tool used to differentiate between neurogenic and myogenic muscle damage. Spontaneous potentials, such as fibrillation potentials and positive sharp waves, arise because of increased muscle fiber sensitivity to acetylcholine 2–3 weeks after the loss of neural innervation (25). Needle electromyography revealed neurogenic damage in the left vastus medialis, whereas no significant abnormalities were observed in the left vastus lateralis. The patient did not experience a significant decrease in muscle strength in the left lower limb because the femoral nerve neurofibroma was located on a branch of the femoral nerve that innervates the quadriceps rather than on the main trunk. Therefore, the remaining normal nerve sections were able to compensate for their primary function. Postoperative sensory loss in the medial aspect of the lower leg corresponds closely to the distribution of the saphenous nerve (26). Based on the surgical approach and intraoperative manipulation, together with electromyography findings, it is presumed that the saphenous nerve may have been stretched or injured during the procedure, resulting in the observed sensory deficits.

Isolated neurofibromas of the femoral nerve are uncommon, with only a limited number of cases reported to date. Most reported cases present with a palpable mass or localized swelling, often accompanied by pain or neurological deficits, depending on the extent of nerve involvement. In contrast, cases presenting primarily with muscle atrophy and pain without an obvious mass are uncommon and may lead to delayed or misdiagnosis (27–30). Recent studies have emphasized the importance of combining imaging modalities with electrophysiological assessments to improve diagnostic accuracy and facilitate early intervention (31).

This case is noteworthy for several reasons. First, the initial presentation of knee pain and medial thigh discomfort closely mimicked lumbar spine pathology, which may have led to misdiagnosis, particularly in the presence of concurrent lumbar disc degeneration. Second, unlike typical cases presenting with a palpable mass, this patient primarily exhibited muscle atrophy and neurogenic pain without an obvious mass, further increasing diagnostic difficulty. Third, the diagnosis was established using a combination of electrophysiological and multimodal imaging findings, highlighting the importance of integrating EMG with ultrasound and MRI for the evaluation of atypical peripheral nerve lesions. Finally, detailed follow-up findings, including postoperative sensory deficits and gradual functional recovery, provide valuable insights into the clinical course and prognosis of femoral nerve neurofibromas.

Despite these findings, this study has limitations. Owing to the unavailability of detailed surgical documentation from the patient, specific technical details regarding nerve grafting and flap reconstruction could not be fully retrieved. This may have limited the completeness of the surgical description in this report.

This case highlights the importance of considering peripheral nerve tumors in patients presenting with unexplained lower limb pain and muscle atrophy, particularly when lumbar imaging findings are insufficient to explain clinical symptoms (32). Although most neurofibromas initially presented as palpable masses, the femoral nerve neurofibroma in this patient was not detectable on physical examination and instead manifested as muscle pain and atrophy, further increasing the risk of misdiagnosis. Early recognition and comprehensive evaluation, including electrophysiological and imaging studies, are essential to avoid misdiagnosis and ensure timely intervention.

## 4 Patient perspective

The patient reported that the pain in the left knee and medial thigh significantly affected his daily activities before treatment. After surgical intervention, he noticed gradual improvement in muscle atrophy and relief of pain. Although some sensory loss persisted in the medial aspect of the lower leg in the early postoperative period, the patient reported progressive recovery over time and expressed overall satisfaction with the treatment outcome.

## Data Availability

The original contributions presented in the study are included in the article/supplementary material. Further inquiries can be directed to the corresponding author.
